# Multi-Time-Scale Optimal Scheduling Strategy for Marine Renewable Energy Based on Deep Reinforcement Learning Algorithm

**DOI:** 10.3390/e26040331

**Published:** 2024-04-14

**Authors:** Ren Xu, Fei Lin, Wenyi Shao, Haoran Wang, Fanping Meng, Jun Li

**Affiliations:** School of Information and Automation, Qilu University of Technology, Jinan 250353, China; 10431210547@stu.qlu.edu.cn (R.X.); 10431210522@stu.qlu.edu.cn (W.S.); 10431221017@stu.qlu.edu.cn (H.W.); 10431220992@stu.qlu.edu.cn (F.M.); rogerjunli@sdu.edu.cn (J.L.)

**Keywords:** deep reinforcement learning, energy scheduling, energy forecast, entropy value, multi-energy complementarity

## Abstract

Surrounded by the Shandong Peninsula, the Bohai Sea and Yellow Sea possess vast marine energy resources. An analysis of actual meteorological data from these regions indicates significant seasonality and intra-day uncertainty in wind and photovoltaic power generation. The challenge of scheduling to leverage the complementary characteristics of various renewable energy sources for maintaining grid stability is substantial. In response, we have integrated wave energy with offshore photovoltaic and wind power generation and propose a day-ahead and intra-day multi-time-scale rolling optimization scheduling strategy for the complementary dispatch of these three energy sources. Using real meteorological data from this maritime area, we employed a CNN-LSTM neural network to predict the power generation and load demand of the area on both day-ahead 24 h and intra-day 1 h time scales, with the DDPG algorithm applied for refined electricity management through rolling optimization scheduling of the forecast data. Simulation results demonstrate that the proposed strategy effectively meets load demands through complementary scheduling of wave power, wind power, and photovoltaic power generation based on the climatic characteristics of the Bohai and Yellow Sea regions, reducing the negative impacts of the seasonality and intra-day uncertainty of these three energy sources on the grid. Additionally, compared to the day-ahead scheduling strategy alone, the day-ahead and intra-day rolling optimization scheduling strategy achieved a reduction in system costs by 16.1% and 22% for a typical winter day and a typical summer day, respectively.

## 1. Introduction

In recent years, with the increasingly severe problem of energy depletion, the development of renewable energy has gradually become the key to solving the energy crisis. Due to the volatility of renewable energy, the power system needs sufficient flexibility to balance the difference between power supply and demand [1]. According to research, the proportion of renewable energy is expected to increase to 31% by the year 2035 [2]. Therefore, effective dispatch for renewable energy sources, particularly taking into account their complementarities, is essential to ensure distribution network stability.

Currently, numerous achievements have been made in the research on the optimal scheduling of renewable energy resources. Day-ahead scheduling involves energy forecasting, with the authors of [3,4,5,6,7] focusing on wind power prediction and the authors of [8,9,10,11] on photovoltaic power prediction. In [12], the authors utilized actual operational data from the load system to perform load forecasting on the integrated energy system of industrial parks using deep learning algorithms, and [13] concentrates on load forecasting. However, these studies predominantly forecast from a single perspective, and simultaneous predictions of both the generation and consumer sides in the system could further enhance grid stability. Dahmani et al. [14] analyzed the reliability of offshore wind power, providing valuable insights. In an earlier work, ref. [15] introduces an integrated multi-energy complementary coordination scheduling method. A storage system control model (ESSCM) is proposed in [16] for the wind and solar hybrid combined storage system scenario to facilitate the synergistic operation of wind and photovoltaic (PV) power generation in a combined system, thus maximizing the benefits of the combined system in the electricity market. Zhang et al. [17] present a day-ahead scheduling model for an industrial power system integrating wind power and multiple types of storage, proving that the introduction of storage devices can reduce the occurrence of wind curtailment and enhance system flexibility. The authors of [18] offer a solution for coordinated optimal day-ahead scheduling in a hybrid thermal–wind–photovoltaic power generation system including an energy storage system (ESS), aimed at minimizing the total generation cost and suppressing frequent changes in the charging and discharging states of the ESS. Reddy et al. [19] propose an optimized scheduling strategy for a battery–thermal–wind–photovoltaic generation system considering the impact of uncertainties in wind, solar photovoltaic, and load forecasting. These scheduling schemes in [14,15,16,17,18,19] focus on the combination of single or dual types of energy sources. The authors of [17,18] do not consider the cost issues of storage systems. Moreover, different maritime areas have distinct climatic characteristics, and relying solely on photovoltaic and wind energy may not always meet the users’ electricity demands. As an emerging energy source, wave energy, with its high predictability and abundant kinetic energy conversion potential, is attracting increasing attention. This paper aims to propose a multi-energy complementary system suitable for the Bohai and Yellow Sea areas.

The Bohai Sea and Yellow Sea, located in the eastern maritime region of China, are geographically connected and significantly influenced by the monsoon climate in terms of their climatic characteristics. For photovoltaic power generation, this area enjoys long sunshine hours and high solar radiation intensity in the summer, while the winter has relatively shorter sunshine hours and less intense solar radiation. Regarding wind power generation, influenced by monsoons, the region is dominated by northerly winds in winter, characterized by stable and strong wind conditions; in summer, it shifts to predominantly southerly winds, which are less stable and weaker. Wave energy in this area is primarily driven by wind waves, exhibiting distribution characteristics similar to the wind field, with higher waves and northerly directions in winter, and lower waves with southerly directions in summer. During the day, the peak production period of photovoltaic power generation is utilized to meet the high electricity demand during peak daytime hours, with wind and wave power generation serving as supplements. Since photovoltaic power generation does not produce electricity at night, wind and wave power generation can provide power, especially on nights with high wind speeds, where wind power generation can play a more significant role. An earlier work [20] assessed the complementary system of wind, photovoltaic, and wave energies in the Yellow Sea and Bohai Sea areas, demonstrating the rationality of their complementarity. Based on the rationale of wind–photovoltaic–wave complementarity, this paper proposes a day-ahead and intra-day multi-time-scale rolling optimization scheduling model that considers the integration of offshore wind energy, wave energy, and offshore photovoltaic energy. This multi-energy integration approach can more effectively utilize the monsoon characteristics of the area to enhance grid stability.

The main work is as follows:

- Based on meteorological data from the Bohai Sea and Yellow Sea areas, we conducted an analysis of annual electricity production, which included the temporal distribution, Kendall coefficient, and entropy value. We discovered that the integration of wave energy could effectively complement wind energy, enhancing the complementarity between wind and solar photovoltaic energy sources.
- We utilized a CNN-LSTM neural network to predict the power generation of three types of renewable energy sources 24 h and 1 h in advance, with time scales of 1 h and 10 min, respectively. Employing CNN-LSTM for predictions at 1 h and 10 min scales captures the short-term and long-term patterns of renewable energy output. This multi-time-scale approach aids in more accurately understanding and predicting the power generation of renewable energy sources, thereby facilitating more effective planning and scheduling of resources.
- We formulated the day-ahead and intra-day energy scheduling problem as a Markov Decision Process (MDP) model. Within this MDP model, we used a DRL algorithm to find the optimal scheduling strategy, adjusting the reward function and state space to better accommodate the rolling optimization scheduling problem discussed in this paper.

In Section 2, we introduce the system models and the cost functions of each unit. In Section 3, we present the day-ahead and intra-day rolling optimization scheduling strategy and the CNNLSTM-DDPG algorithm. Section 4 is dedicated to simulation analysis, and Section 5 concludes the discussion.

## 2. System Model

### 2.1. Energy Complementarity Analysis

**Figure 1:**
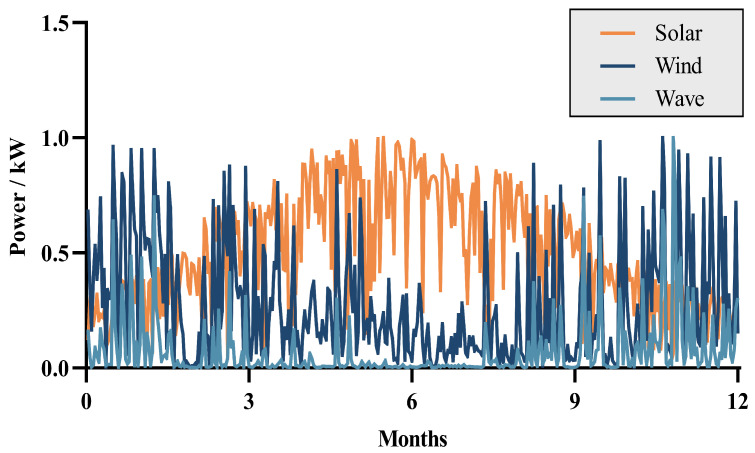
The annual power generation from marine renewable energy sources.

We collected meteorological data from the maritime areas surrounding Weihai City in Shandong Province, China, for the period from 1 January to 31 December 2019. Through the use of power conversion formulas, we calculated the annual power generation. For ease of analysis, the calculated data were normalized, as shown in Figure 1.

The generating power of a wind turbine (WT) is directly related to the wind speed, and the equation for the output power of a wind turbine is expressed as [21]

(1) $$P_{WT}=\left\{\begin{array}{ll} 0, & v_{i}<v_{in}\;or\;v_{out}<v_{i}\\ P_{Rated}\frac{v_{i}^{3}-v_{in}^{3}}{v_{r}^{3}-v_{in}^{3}}, & v_{in}<v_{i}<v_{r}\\ P_{Rated}, & v_{r}<v_{i}<v_{out} \end{array}\right.,$$

where $v_{in}$ is the cut-in wind speed and $v_{out}$ is the cut-out wind speed. When the wind speed exceeds the cut-out wind speed, the wind turbine will stop generating electricity to protect the turbine blades. When the wind speed ($v_{i}$) is between the rated wind speed ($v_{r}$) and the cut-out speed ($v_{out}$), the wind turbine outputs its rated power.

When calculating the power output of a wind turbine ($P_{WT}$) based on wind speed, it is necessary to convert the wind speed according to the height of the wind turbine (WT), using the following conversion formula [22]:

(2) $$v=v_{m}{\left(\frac{h}{h_{m}}\right)}^{z},$$

where v is the wind speed at the hub height (h) of the WT, $v_{m}$ is the wind speed measured at the height, $h_{m}$, and z is a function of atmospheric stability and ocean surface characteristics, with a value of 0.11 [20].

The performance of photovoltaic (PV) power generation is primarily influenced by solar radiation and ambient temperature. The calculation formula is as follows [22]:

(3) $$P_{PV}=P_{Rated}\frac{R_{i}}{R_{STC}}\left[1+k\left(T_{cell}-T_{STC}\right)\right]$$

In the formula, $T_{cell}$ represents the working temperature of the photovoltaic panel, $T_{STC}$ and $R_{STC}$ are the temperature and radiation values measured under standard conditions, $R_{i}$ is the current radiation, $P_{Rated}$ is the rated power, and k is the temperature conversion coefficient, set at −3.7%.

For the wave energy output model, this paper employs an oscillating buoy as the wave energy converter (WEC), represented as [23,24]

(4) $$P_{Wave}=\eta \frac{\rho g^{2}H_{s}^{2}T_{e}}{32\pi }L_{width}$$

In the formula, η represents the energy conversion efficiency, $L_{width}$ represents the width of the wave captured, $H_{s}$ and $T_{e}$, respectively, represent the effective wave height and effective wave period, and ρ and g are the density of seawater and the acceleration due to gravity, respectively.

The Kendall coefficient (τ) is an indicator used to evaluate the correlation between two sets of data. Through the Kendall coefficient, the complementary potential between different energy sources can be analyzed [19]. When 0<τ<1, the two energy sources are positively correlated, indicating that they have a similar increase or decrease relationship and lack complementarity; when −1<τ<0, the two energy sources are negatively correlated, indicating that they have opposite increase or decrease relationships and possess complementarity. Table 1 shows the complementarity among photovoltaic, wave energy, and wind energy sources.

**Figure 2:**
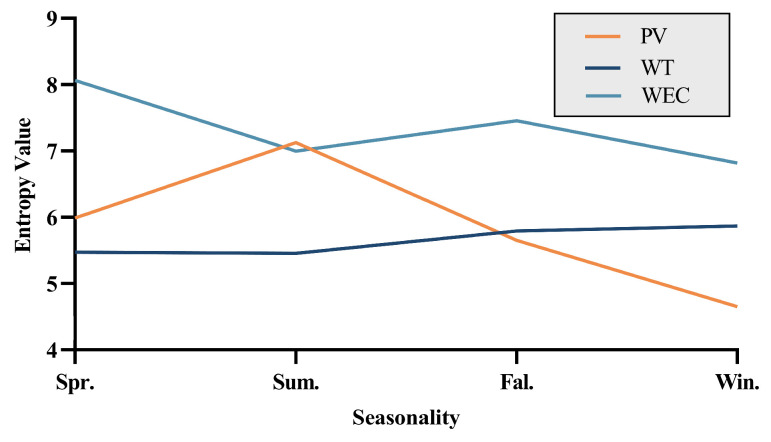
The entropy values from marine renewable energy sources.

By evaluating the annual electricity production entropy of three energy sources, their uncertainty and variability can be quantified. Periods with higher entropy values indicate significant fluctuations in electricity production for the corresponding energy source, signifying increased uncertainty; conversely, lower entropy values suggest more stable production levels. As illustrated in Figure 2, photovoltaic (PV) and wave energy conversion (WEC) exhibit an approximate inverse relationship in entropy values throughout the year, suggesting that these two energy sources can complement each other in facing changes and instability. Meanwhile, wind turbine (WT) entropy values remain relatively stable over the year, contributing positively to enhancing the stability of grid operations.

From Figure 1 and Table 1, it is evident that on a seasonal time scale, wind energy and photovoltaic energy exhibit good complementarity, while wave energy is correlated with wind energy and also has good complementary potential with photovoltaic energy. $\tau_{(WT+WEC)-PV}$ in Table 1 shows that after integrating the WEC into the WT-PV system, the Kendall coefficient changes from −0.3649 to −0.4106, indicating an enhancement in complementarity. Therefore, the inclusion of wave energy can effectively compensate for the shortcomings of wind energy.

Based on a comprehensive analysis that considers the temporal distribution, Kendall coefficient, and entropy values of the three energy sources, we conclude that it is reasonable to implement complementary scheduling of wind, solar, and wave energy in the test marine area.

### 2.2. Microgrid Generation Model

**Figure 3:**
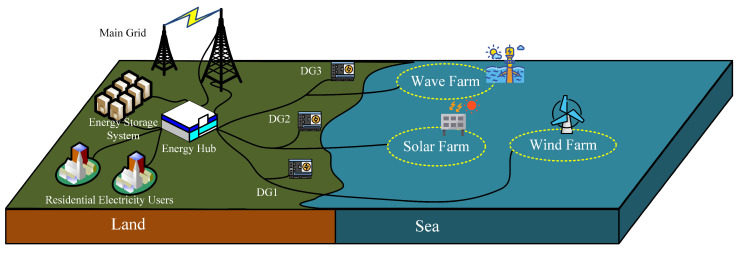
The microgrid generation model.

Based on the aforementioned analysis, we considered a wind–solar–wave multi-energy complementary system, as shown in Figure 3. This system consists of three microgrids, each equipped with renewable energy generation facilities and Distributed Generation (DG) devices as backup power sources. In this system, the generation of various energy sources is subject to environmental conditions, leading to uncertainties and volatility. To balance power generation with load demand, surplus electricity can be stored in the energy storage system for future use. Moreover, when the renewable energy generation exceeds load demand, this surplus electricity can also be sold through energy transactions with the main grid, thereby achieving optimal energy allocation and maximizing economic benefits.

For electricity pricing, this paper adopts time-of-use (TOU) pricing. The year is divided into heating and non-heating periods, and the day is divided into peak and off-peak hours. The non-heating period spans from 1 April to 31 October, and the heating period from November 1 to March 31 of the following year. During the non-heating period, peak hours are from 8:00 to 22:00, and off-peak hours are from 22:00 to 8:00 (the next day); during the heating period, peak hours are from 8:00 to 20:00, and off-peak hours are from 20:00 to 8:00 (the next day). The electricity price data were obtained from the official website of the State Grid Shandong Electric Power Company, and are shown in Table 2.

### 2.3. Cost Function

The goal of this paper is to achieve system cost minimization through microgrid scheduling optimization while fulfilling load demands. Consequently, we take into account the operational costs of each microgrid unit, transaction costs, and penalty costs simultaneously. To encourage the system to prioritize the use of renewable energy to satisfy the load demand, we set the selling price of electricity from the microgrid at 50% of the current electricity rate.

The operating costs of DG units can typically be approximated by a quadratic equation [19] due to the non-linear relationship between the cost and the generated electricity. This relationship encompasses fixed costs, costs that change linearly, and costs that are proportional to the square of the generated electricity.

(5) $$C_{i,t}^{DG}=a_{i}{\left(P_{i,t}^{DG}\right)}^{2}+b_{i}\left(P_{i,t}^{DG}\right)+c_{i},i\in \left\{1,2,3\right\},$$

To ensure that the operating costs of DG units are lower than the cost of purchasing electricity, the specific values of a, b, and c are shown in Table 3.

The cost of trading electricity in the system is expressed as

(6) $$C_{t}^{E}=p_{t}(P_{t}^{Buy}-P_{t}^{Sell}),$$

where $p_{t}$ is the electricity price at the current moment, and $P_{t}^{Buy}$ and $P_{t}^{Sell}$ are the amounts of electricity to be bought and sold, respectively.

The operating cost of renewable energy equipment is expressed as

(7) $$C_{t}^{R}=C_{t}^{WT}+C_{t}^{PV}+C_{t}^{WEC}=(k_{WT}P_{t}^{WT}+k_{PV}P_{t}^{PV}+k_{WEC}P_{t}^{WEC})\Delta t$$

k is the linear coefficient

The operating cost of the energy storage system (ESS) is expressed as

(8) $$C_{t}^{ESS}=k_{ESS}\left|P_{t}^{swap}\right|\Delta t$$

The variable $P_{t}^{swap}$ represents the charging and discharging power of the ESS. If $P_{t}^{swap}$ is less than 0, the device is discharging; otherwise, the device is charging.

When condition $P_{i,t}^{DGdispatch}+P_{t}^{Rdispatch}+P_{t}^{ESSdisch\arg e}+P_{t}^{Buy}<P_{t}^{Load\_Actual}$ occurs and the power demand of users cannot be met during time period t, this is referred to as a power deficit situation. The sum on the left side of the inequality represents the scheduled power $P_{t}^{dispatch}$. We set the penalty cost for power shortages as

(9) $$C_{t}^{deficit}=k_{deficit}P_{t}^{deficit}=k_{deficit}(P_{t}^{Load\_Actual}-P_{t}^{dispatch})$$

When condition $P_{i,t}^{DGdispatch}+P_{t}^{Rdispatch}+P_{t}^{ESSdisch\arg e}+P_{t}^{Buy}>P_{t}^{Load\_Actual}$ occurs, the scheduled power $P_{t}^{dispatch}$ during time period t overflows, resulting in curtailment situation. We set the penalty cost for curtailment as

(10) $$C_{t}^{abandon}=k_{abandon}P_{t}^{abandon}=k_{abandon}(P_{t}^{dispatch}-P_{t}^{Load\_Actual})$$

The detailed parameters are shown in Table 4.

## 3. CNNLSTM-DDPG Algorithm

### 3.1. Day-Ahead and Intra-Day Rolling Optimization Scheduling Strategy

**Figure 4:**
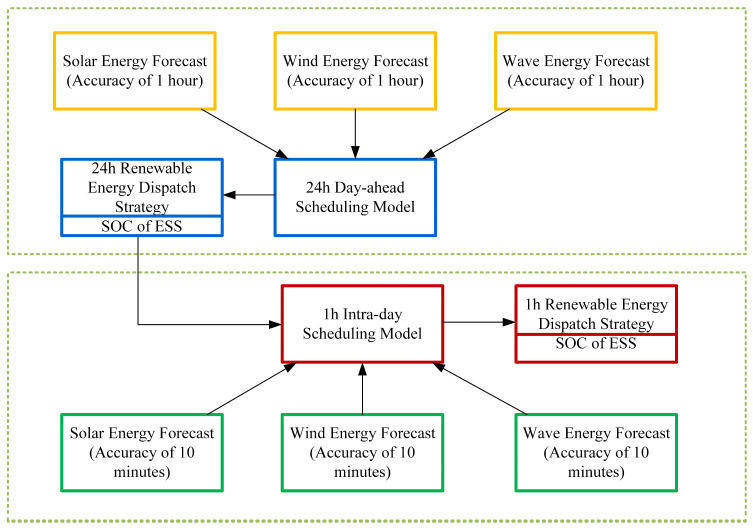
Day-ahead and intra-day optimization scheduling strategy.

The day-ahead and intra-day rolling optimization scheduling strategy is shown in Figure 4.

The foundation of rolling optimization is the day-ahead and intra-day forecasting. Day-ahead forecasting utilizes the CNN-LSTM network with a 1 h time step to predict the power generation from photovoltaics, wave energy, and wind turbines, as well as load demand, 24 h in advance. Intra-day forecasting, with a 10 min time step, predicts renewable energy generation and load demand 1 h ahead.

In the day-ahead optimization phase, based on the day-ahead forecast results, a deep reinforcement learning algorithm is used to formulate an hourly scheduling plan for the ESS, DG, and the main grid, at 1 h intervals, 24 h in advance. The day-ahead objective function can be represented as

(11) $$\min\sum_{i}^{N}\sum_{t}^{T}\left(C_{t,i}^{DG}+C_{t}^{R}+C_{t}^{ESS}+C_{t}^{E}\right),$$

(11a) $$s.t.\;\sum_{t}^{T}\left(\sum_{i}^{N}P_{i,t}^{DG}+P_{t}^{R}+P_{t}^{swap}+P_{t}^{Buy}-P_{t}^{Sell}\right)=\sum_{t}^{T}P_{t}^{load},$$

(11b) $$P_{\min}^{swap}\leq P_{t}^{swap}\leq P_{\max}^{swap},\forall t\in T,$$

(11c) $$SOC_{\min}^{ESS}\leq SOC_{t}\leq SOC_{\max}^{ESS},\forall t\in T,$$

(11d) $$SOC_{t}=SOC_{t-1}+\eta_{ESS}P_{t}^{swap}\Delta t,\forall t\in T,$$

where (11a) ensures the balance of system power; (11b) indicates the maximum charging and discharging power of the energy storage system; (11c) limits the State of Charge (SOC) of the energy storage system; and (11d) represents the SOC update strategy.

Intra-day optimization is based on the output of day-ahead optimization, adjusting the dispatch plan according to the deviations between intra-day forecasts and day-ahead forecasts. To maximize system economy, intra-day optimization tries to avoid changes to the scheduling plans of DG, ESS, and other units, focusing instead on re-planning the deviated wind, solar, and wave energy to perform peak shaving and valley filling. Adjustments to other units’ scheduling plans are made only when the aforementioned operations cannot meet the load demand. The cost of intra-day optimization considers the impact of day-ahead forecast deviations on system costs, represented as

(12) $$C_{t}^{IN}=k_{WT}(P_{t,WT}^{Day-ahead}-P_{t,WT}^{Intra-day})+k_{WEC}(P_{t,WEC}^{Day-ahead}-P_{t,WEC}^{Intra-day})+k_{PV}(P_{t,PV}^{Day-ahead}-P_{t,PV}^{Intra-day})$$

Additionally, to encourage intra-day optimization to schedule only the energy with forecast deviations without altering the day-ahead decisions, we introduce an additional constraint:

(13) $$C_{t}^{R}<C_{t}^{ESS}<C_{i,t}^{DG}<C_{t}^{E}$$

The objective function is updated as

(14) $$\min\sum_{i}^{N}\sum_{t}^{T}\left(C_{t,i}^{DG}+C_{t}^{R}+C_{t}^{ESS}+C_{t}^{IN}+C_{t}^{E}\right)$$

### 3.2. CNNLSTM-DDPG Algorithm

Despite the discernible patterns exhibited by renewable energy sources, they also possess characteristics of uncertainty and volatility. The fundamental cause of these phenomena is the variability of weather conditions. Sudden changes in weather can increase the challenges of forecasting, as simple prediction models may fail to adequately capture such abrupt changes, leading to decreased forecasting accuracy. In the scenario considered in this paper, high accuracy is required to support the stability of the power supply in the scheduling system. Component-based forecasting is an excellent prediction method, as it allows for a better understanding of the factors behind renewable energy forecast results, but it inevitably increases the complexity of the process by requiring the integration of predictions from various components. Regarding neural network prediction models, a vast body of research has validated their reliability. Compared to component-based forecasting, neural network models may seem more complex at first glance, but they can autonomously capture and learn from complex relationships within the data, avoiding the need for result integration, and offering considerable accuracy in predictions. Considering both efficiency and accuracy, we have chosen to use neural network models for forecasting. For such long-term sequential data, CNN-LSTM stands out as an excellent choice.

The CNN-LSTM network is a hybrid neural network model that combines Convolutional Neural Networks (CNNs) and Long Short-Term Memory (LSTM) networks. This structure leverages the powerful capability of CNNs in processing spatial features along with the advantages of LSTM in handling time-series data, making it particularly suitable for the complex and temporal correlation task of predicting renewable energy generation in this paper.

Wind, wave, and photovoltaic energies in the Bohai and Yellow Sea regions are influenced by weather conditions, seasonal changes, and other factors. CNNs can effectively extract spatial features, while LSTM excels at capturing the dynamic changes in these data over time. Utilizing both CNNs and LSTM allows for a more comprehensive understanding and use of these temporal correlation features, thereby enhancing prediction accuracy. And the CNN-LSTM model is capable of efficiently processing datasets by automatically extracting important features, thereby saving time and enhancing efficiency.

This paper employs a CNN-LSTM network for 24 h day-ahead forecasting and 1 h intra-day forecasting of renewable energy generation data. When forecasting 24 h in advance, the CNN-LSTM network can analyze the daily variation trends of energy production based on past data, outputting future predictions for the next 24 h on an hourly time scale. This provides crucial information for the energy dispatch and management of the power grid. For predictions within an hour, the CNN-LSTM model can capture short-term fluctuations in energy production more accurately, outputting predictions for the next hour on a 10 min time scale. This is vital for adjusting the grid’s immediate load and optimizing energy distribution. The combination of day-ahead forecasting and intra-day forecasting can effectively reduce the impact of renewable energy instability on the distribution grid.

Based on the forecast data from the CNN-LSTM network, the energy scheduling optimization problem can be formulated as a Markov Decision Process (MDP) decision model, which can then be solved using deep reinforcement learning algorithms. This model is represented by a quintuple (S,A,P,R,γ), where S represents the state space, A represents the action space, P represents the state transition probability, R represents the reward function, and γ represents the discount factor. In this paper, the state space consists of the power generation of renewable energy, power demand, and the charging/discharging status of batteries, which is $s_{t}=(P_{t}^{R},P_{t}^{Load},SOC_{t},p_{t})$. Based on the state of the microgrid system at time t, the action space for the microgrid is defined as $a_{t}=(P_{i,t}^{DG},P_{t}^{swap},P_{i,t}^{R},P_{t}^{Sell},P_{t}^{Buy})$. P is the transfer probability of transferring from the current state, $s_{t}$, to the next state, $s_{t+1}$, after executing the current action, $a_{t}$. The deterministic policy, π:S→P(A), is defined as the mapping of received states to actions. Different actions explored in the environment will receive different rewards. The goal of reinforcement learning is to use the reward function as a guide to discover the action that maximizes rewards as the optimal solution to the optimization problem. The objective of this paper is to minimize the operational costs of the system. The day-ahead scheduling reward function is represented as follows:

(15) $$R_{t,Day-ahead}(s_{t},a_{t})=-\min\sum_{i}^{N}\sum_{t}^{T}\left(C_{t}^{DG}+C_{t}^{R}+C_{t}^{ESS}+C_{t}^{E}\right)$$

For intra-day optimization, the reward function is adjusted accordingly:

(16) $$R_{t,Intra-day}(s_{t},a_{t})=-\min\sum_{i}^{N}\sum_{t}^{T}\left(C_{t}^{DG}+C_{t}^{R}+C_{t}^{ESS}+C_{t}^{IN}+C_{t}^{E}\right)$$

The authors of [25,26] have effectively addressed the issue of cost reduction by incorporating deep reinforcement learning (DRL) into the energy dispatch problem of energy systems. The Deep Deterministic Policy Gradient (DDPG) algorithm has garnered attention for its effective handling of continuous action space problems [27,28]. This algorithm combines the representational capabilities of deep learning with the decision optimization techniques of policy gradient methods, adopting a variant of the Actor–Critic architecture, implemented through deep neural networks to approximate both policy and value functions [29]. The actor network directly maps states to deterministic actions, while the Critic network evaluates the expected return for given states and actions [30]. Additionally, DDPG incorporates an experience replay mechanism, storing past transitions (states, actions, rewards, and new states) for reuse during training, thus reducing correlations between samples and enhancing learning stability. To further stabilize the learning process, DDPG also employs target network technology, setting up target networks for both the Actor and Critic and slowly updating their parameters, which helps to mitigate the issue of moving targets.

**Figure 5:**
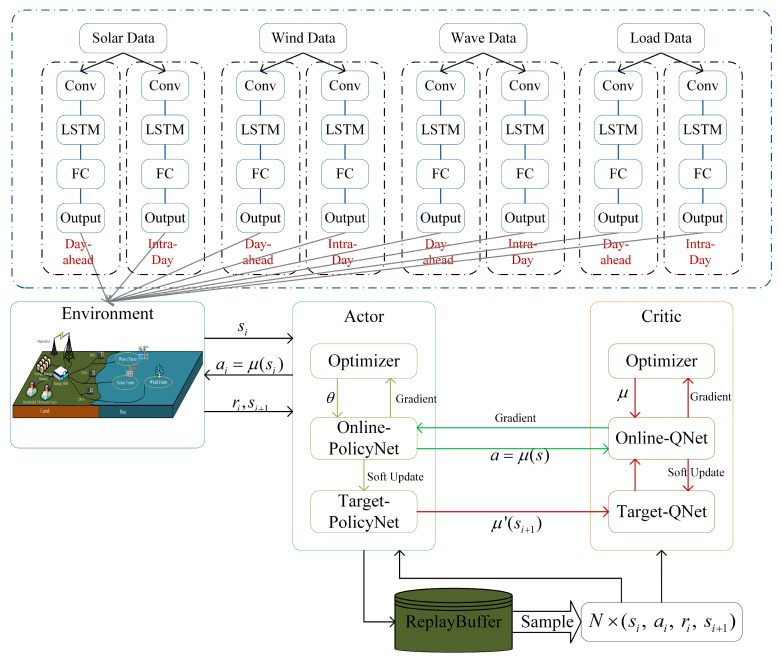
CNNLSTM-DDPG algorithm.

The flow of the algorithm is shown in Figure 5 and Algorithm 1.

**Algorithm 1:** DDPG
**1.**
**Initialize:** The Critic networks $Q(s,a|\theta^{Q})$ and Actor network $\mu (s|\theta^{\mu })$; the weights are $\theta^{Q}$ and $\theta^{\mu }$. The Critic target networks $Q\prime (s,a|\theta^{Q})$ and Actor target network μ′ have weights $\theta^{Q\prime }\leftarrow \theta^{Q}$ and $\theta^{\mu \prime }\leftarrow \theta^{\mu }$ The experience playback buffer (R) has size n. Empty the experience playback buffer (R).
**2.**
**for** episode = 1, 2, …, T **do**
**3.**
Reset the simulation parameters of the energy dispatch system to obtain the initial observation state, $s_{1}$.
**4.**
    **for** i = 1, 2, …, I **do**
**5.**
    Normalize state $s_{i}$ to $s_{i}\prime$.
**6.**
    Obtain Actor network action $a_{i}$ and noise $n_{i}$:                                                        $a_{i}=\min(\max(\mu (s_{i}\prime |\theta^{\mu })+n_{i},-1),1)$
**7.**
    Execute action $a_{i}$, obtain the reward, $r_{i}$, and observe the new state, $s_{i+1}$.
**8.**
    Store transmission $\left(s_{i}\prime ,a_{i},r_{i},s_{i+1}\prime \right)$ to the Replay Buffer (R).
**9.**
    Select a batch of transition $\left(s_{j}\prime ,a_{j},r_{j},s_{j+1}\prime \right)$ from R, ∀j=1,2,…,I
**10.**
    Calculate $Q_{t\arg et,j}=r_{j}+\gamma Q\prime (s_{j+1},a_{j}\prime |\theta^{Q\prime })$
**11.**
    Update the Critic network parameters $\theta_{j}^{Q}$ based on the mean square     loss function:                                                        $L(\theta^{Q})=\frac{1}{N}\sum_{N=1}^{N}\left({\left(Q_{t\arg et,j}-Q(s_{j}\prime ,a_{j}|\theta^{Q})\right)}^{2}\right)$.
**12.**
    Update the Actor network using the stochastic policy gradient:                                                        $\nabla_{\theta \mu }J\approx \frac{1}{N}\sum_{j}\nabla_{a}Q(s,a|\theta^{Q}){|}_{s=sj,a=\mu (sj)}\nabla_{\theta \mu }\mu (s|\theta^{\mu })|s_{j}$.
**13.**
    Update the target network parameters:                                                        $\theta^{Q\prime }\leftarrow \tau \theta_{i}^{Q}+(1-\tau )\theta_{i}^{Q\prime }$,                                                        $\theta^{\mu \prime }\leftarrow \tau \theta^{\mu }+(1-\tau )\theta^{\mu }$.
**14.**
    **end for**
**15**

**end for**


The initial stage involves day-ahead scheduling optimization, wherein the CNN-LSTM network is utilized to obtain initial value $s_{i}^{Day-ahead}$ for the future 24 h renewable energy generation, power demand, battery State of Charge (SOC), and current electricity price. The Actor network gives the power scheduling plan $a_{i}^{Day-ahead}$ in state $s_{i}^{Day-ahead}$ through $a_{i}^{Day-ahead}=\min(\max(\mu (s_{i}^{Day-ahead}\prime |\theta^{\mu })+n_{i},-1),1)$. The agent, upon executing action $a_{i}^{Day-ahead}$ within the environment, receives rewards, $r_{i}^{Day-ahead}$, and transitions to the next state, $s_{i+1}^{Day-ahead}$. The reward function for day-ahead scheduling is detailed in Equation (15). At this juncture, the Critic network assesses the action value function, $Q(s_{i}^{Day-ahead},a_{i}^{Day-ahead})$, of the current scheduling plan, $a_{i}^{Day-ahead}$, evaluating the plan’s value. Then, the $s_{i+1}^{Day-ahead}$ is fed into both the Target Actor and Target Critic networks. The Target Actor proposes a scheduling plan, $a_{i+1}^{Day-ahead}$, for state $s_{i+1}^{Day-ahead}$, and the Target Critic calculates the maximum Q-value, $Q_{\mathrm{target}}^{Day-ahead}$, for the optimal action of the next state via the Bellman optimality in Equation (17), which is relayed back to the Critic network. The quadruplet $\left(s_{i}^{Day-ahead},a_{i}^{Day-ahead},r_{i}^{Day-ahead},s_{i+1}^{Day-ahead}\right)$ is stored in the experience replay pool (R) for subsequent training.

(17) $$Q_{target,i}^{Day-ahead}=r_{i}^{Day-ahead}+\gamma Q\prime (s_{i+1}^{Day-ahead},\mu \prime (s_{i+1}^{Day-ahead}|\theta^{\mu \prime })|\theta^{Q\prime })$$

Following this, the parameters of both the Actor and Critic networks are updated. The Critic receives the evaluation $Q_{\mathrm{target},i}^{Day-ahead}$ and compares it against its own calculations, $Q(s_{i}^{Day-ahead},a_{i}^{Day-ahead})$, adjusting its objective function $Q_{target,i}^{Day-ahead}-Q(s_{i}^{Day-ahead},a_{i}^{Day-ahead})$ by minimizing the mean squared error loss function (18) to update the Critic network parameters.

(18) $$L(\theta_{j}^{Q})=\frac{1}{N}\sum_{N=1}^{N}\left({\left(Q_{\mathrm{target},j}^{Day-ahead}-Q(s_{j}^{Day-ahead}\prime ,a_{j}^{Day-ahead}|\theta_{j}^{Q})\right)}^{2}\right)$$

The Actor network employs the gradient ascent algorithm, aiming to maximize the expected return estimated by the Critic network, thus updating the network parameters. The policy gradient for updating the Actor network is represented by the following equation:

(19) $$\nabla_{\theta \mu }J\approx \frac{1}{N}\nabla_{a}Q(s,a|\theta_{1}^{Q}){|}_{s=s_{j}^{Day-ahead},a=\mu (s_{j}^{Day-ahead})}\nabla_{\theta \mu }\mu (s|\theta^{\mu }){|}_{s_{j}^{Day-ahead}}$$

Lastly, the target network parameters are updated through a soft update mechanism, employing Equations (20) and (21) to control the update rate of the target network parameters, thereby enhancing learning stability.

(20) $$\theta^{Q\prime }\leftarrow \tau \theta_{i}^{Q}+(1-\tau )\theta_{i}^{Q\prime },$$

(21) $$\theta^{\mu \prime }\leftarrow \tau \theta^{\mu }+(1-\tau )\theta^{\mu }.$$

Thus, the DDPG day-ahead optimization algorithm completes a training iteration. It is important to note that during each training session, the DDPG may randomly draw a batch of experiences from the replay pool (R) to update the network. This process is repeated N times to produce the optimal 24 h day-ahead scheduling plan.

In intra-day scheduling optimization, one hour prior to the actual operation, future one-hour power generation and demand forecasts are obtained from the CNN-LSTM network. Subsequently, the differences between these intra-day forecast results and the pre-established day-ahead scheduling plan are calculated to determine the renewable energy forecast deviation, $P_{t,i}^{R-}$, and the load demand forecast deviation, $P_{t,i}^{L-}$. The state space, $s_{i}^{Intra-day}$, for intra-day scheduling comprises a tuple $\left(P_{t,i}^{R-},P_{t,i}^{L-},SOC_{Day-ahead},p_{t}\right)$ that includes these two forecast deviations, the State of Charge (SOC) coefficient of the energy storage system from the day-ahead plan, and the current electricity price. The network updating process thereafter follows the same procedure as for day-ahead optimization scheduling, with the exception that the reward function is replaced with Equation (16). After N training sessions, the intra-day optimized scheduling plan is output.

## 4. Simulation Analysis

Using the collected data, we created an original dataset. We designated the first three weeks of each month as the training set and the remaining time as the test set. This approach allows the trained algorithm to consider the seasonal variations in renewable energy generation and electricity demand.

### 4.1. Power Generation Forecast

We compared the day-ahead forecast and intra-day forecast results for a typical day and found discrepancies between them. This is because the forecasting error tends to decrease as the time approaches closer to the actual operation. The day-ahead forecast is conducted 24 h before the actual operation, while the intra-day forecast is carried out 1 h prior, utilizing the latest meteorological data and system status information, which may not be as accurate during the day-ahead forecast.

**Figure 6:**
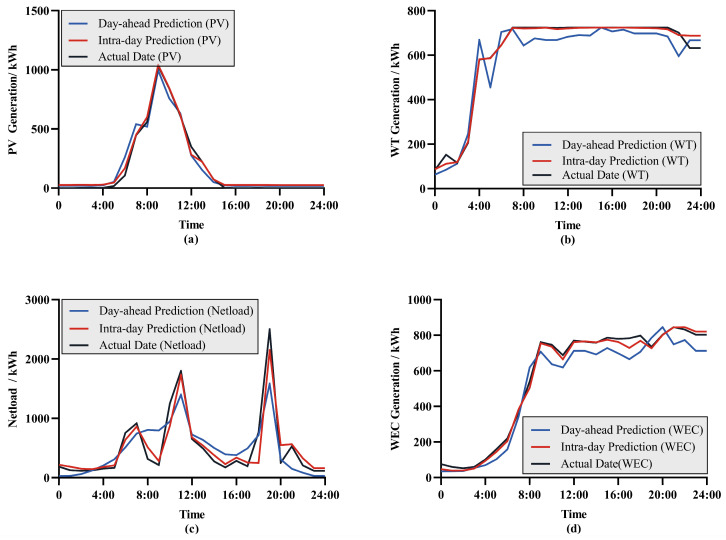
Power forecast results: (**a**) the PV forecast result; (**b**) the WT forecast result; (**c**) the Netload forecast result; (**d**) the WEC forecast result.

The forecast results of a typical day are shown in Figure 6a–d. From Figure 6, it can be observed that the accuracy of day-ahead forecasts is significantly lower compared to intra-day forecasts. This discrepancy can be attributed to two main reasons: (1) The dataset for intra-day forecasting is updated more frequently, with a finer time scale, making it more sensitive to fluctuations in data. (2) The day-ahead forecast involves making predictions 24 steps ahead for a 24 h period, whereas intra-day forecasting involves only 6 steps for predicting 1 h ahead. Naturally, forecasts involving fewer steps tend to be more accurate.

### 4.2. Scheduling Results

The DDPG algorithm was implemented and trained 500 times in Python using Pytorch. We simulated the system’s performance under various monsoon conditions, setting up three renewable energy microgrids continuously to provide power output. The energy storage system (ESS) has a rated capacity of 5000 kW with an efficiency of 0.9. The installed capacities for WEC, WT, and PV are 600 kW, 1000 kW, and 1100 kW, respectively.

**Figure 7:**
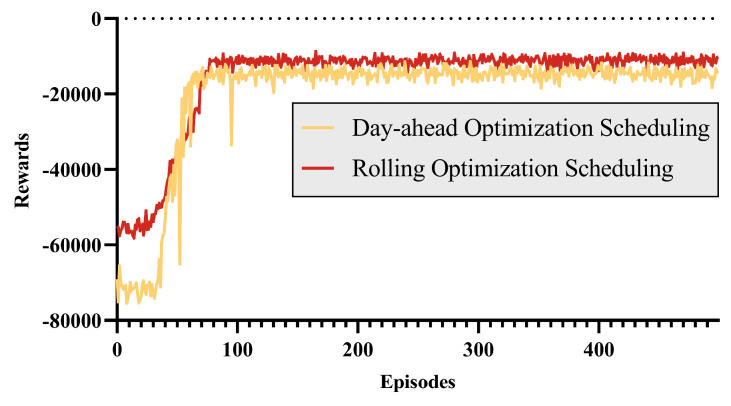
The reward of the DDPG algorithm.

The forecast data were input into both the day-ahead optimization DRL model and the day-ahead and intra-day rolling optimization DRL model, with the reward value convergence curve shown in Figure 7. It can be observed that under the optimization of the DDPG algorithm, the reward gradually increases and stabilizes quickly. The reward for rolling optimization scheduling is greater than that for day-ahead optimization scheduling. This is because the rolling optimization scheduling has a more precise scheduling plan, which reduces penalty costs.

**Figure 8:**
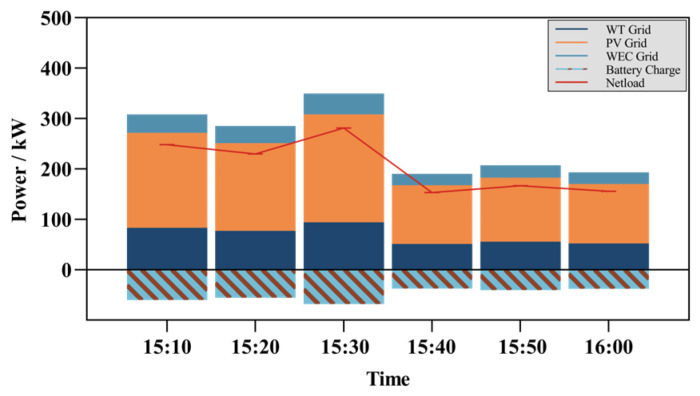
Intra-hour scheduling plan.

As indicated in the hypothetical Figure 8, the intra-day adjustment phase allows for the precise allocation of electricity plans every 10 min, which is not feasible with day-ahead scheduling. It is important to note that the intra-day adjustment phase of rolling optimization scheduling outputs scheduling plans on a 10 min timescale, whereas day-ahead scheduling operates on a 1 h basis. To more intuitively analyze the changes and costs between rolling optimization scheduling and day-ahead optimization scheduling, we standardized the time scale. We aggregated the 1 h intra-day adjustment plans, converting the unit of measurement from power generation capacity (kW) to electricity generation and consumption (kWh).

**Figure 9:**
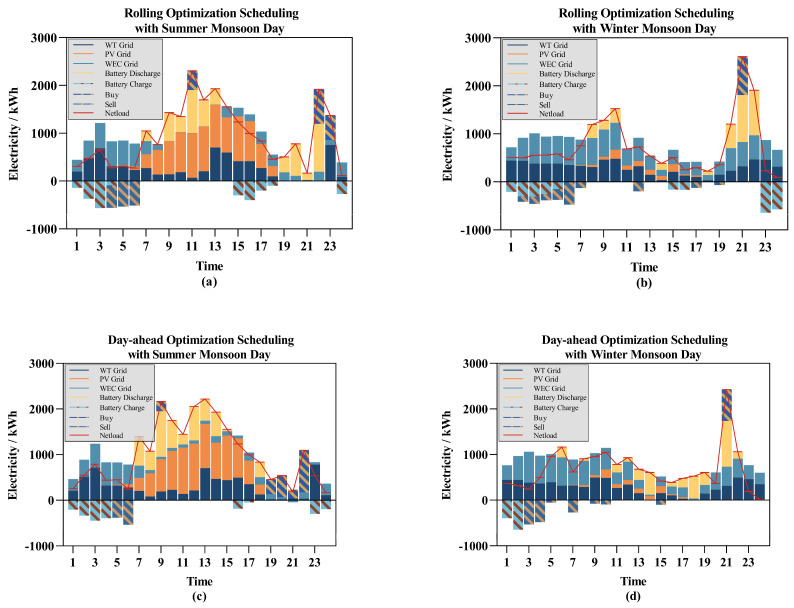
The scheduling result: (**a**) the rolling optimization scheduling with summer monsoon days; (**b**) the rolling optimization scheduling with winter monsoon days; (**c**) the day-ahead optimization scheduling with summer monsoon days; (**d**) the day-ahead optimization scheduling with winter monsoon days.

The scheduling results obtained are shown in Figure 9. The rolling optimization scheduling results are analyzed horizontally. As shown in Figure 9a,b, during a typical summer day, the sunlight conditions are favorable, allowing for stable photovoltaic power generation. Wind resources are concentrated between 01:00 and 7:00 and between 13:00 and 17:00, with the generated surplus energy being stored in the ESS or sold to the main grid at appropriate times. A peak load occurs between 9:00 and 13:00, with the ESS output compensating for the shortage in generation. Notably, on this day, there is almost no renewable energy generation between 19:00 and 22:00, with the ESS playing a key role in maintaining the power supply. Unlike the summer, the typical winter day experiences poor sunlight conditions, resulting in significantly insufficient energy from the photovoltaic system. However, abundant wind resources on this day provide ample wave and wind energy, with the ESS again playing a crucial role during the evening peak load period. The presence of the ESS allows the power supply system to achieve peak shaving and valley filling from the generation side, enhancing system supply stability while avoiding energy waste.

Vertically comparing rolling optimization scheduling with day-ahead optimization scheduling, Figure 9a,c and 9b,d, respectively, show their scheduling plans on a typical summer monsoon day and a typical winter monsoon day. In Figure 9b,d from 11:00 to 19:00, there is a significant difference in the predicted power generation from wave and wind energy. The rolling optimization scheduling algorithm, upon receiving more accurate prediction results, timely adjusted the scheduling plan, reducing system costs. In Figure 9a,c at 11:00–13:00 and 21:00–23:00, and in Figure 9b,d at 7:00–11:00 and 19:00–22:00, there are significant load power fluctuations. The rolling optimization scheduling balanced the power fluctuations by adjusting the renewable energy generation based on prediction deviations and utilizing the energy storage system.

The analysis of climate conditions showed that under different monsoon conditions, the output proportion of various renewable energy sources varies. During the summer monsoon, the region experiences long sunshine hours and high-intensity solar radiation, leading to a high proportion of photovoltaic power generation; wind resources are relatively scarce, resulting in lower proportions of wind and wave energy. During the winter monsoon, when the region experiences high wind speeds and stable wind directions, wind and wave energy generation contribute more to the output.

With an analysis of electricity prices, the inclusion of the energy storage system significantly contributes to reducing the system’s electricity purchasing costs. Observing the State of Charge (SOC) of the energy storage system, it is evident that when the electricity prices are high during the day, the system minimizes electricity purchases from the main grid by releasing the stored energy from the ESS to the maximum extent.

**Figure 10:**
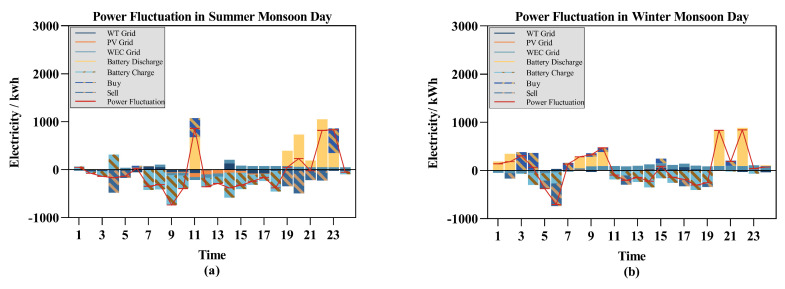
Power fluctuation on different typical days: (**a**) the result of summer monsoon days; (**b**) the result of winter monsoon days.

The subsequent analysis focuses on how intra-day optimization adjustments address the issue of power fluctuations. Here, “power curtailment” and “power deficit” specifically relate to the dispatch plan, not the overall power supply system. This distinction is crucial because, although the integration of energy storage systems and the main grid can dynamically balance power fluctuations to prevent system-wide power curtailment and power deficit, significant deviations in the dispatch plan can lead to substantial short-term overload stress on the equipment. The power curtailment and power deficit in question result from power fluctuations caused by dispatch deviations due to forecast inaccuracies. The objective of the proposed rolling optimization scheduling algorithm is to significantly reduce the occurrence of these issues, thereby lowering costs and enhancing the stability of the system. Figure 10 illustrates the role of the rolling optimization scheduling strategy in mitigating power fluctuations within the day-ahead scheduling plan, detailing how intra-day adjustment plans counteract the effects of power fluctuations, thereby mitigating instances of power curtailment and power deficit.

In Figure 10a, during the 9:00 time slot, the power fluctuation is less than 0, indicating that a power deficit occurred in the day-ahead scheduling plan for that period. In the 7:00–10:00 time slot in Figure 10b, the power fluctuation is greater than 0, indicating a need for more electricity, hence a power curtailment event. Rolling scheduling optimization mitigates power fluctuations by adjusting the supply conditions of each generation unit and the charging/discharging states of storage units in a timely manner.

### 4.3. Cost Analysis

Earlier, we mentioned the potential of wave energy to enhance the complementary nature of the system’s energy resources, with a theoretical analysis provided. Next, we aim to validate the impact of wave energy generation on the system through simulation experiments. In these experiments, we excluded the wave energy generation microgrid. To isolate the variable, the installed capacity of the WT was increased to 1600 kW, with all other equipment specifications remaining as before.

**Figure 11:**
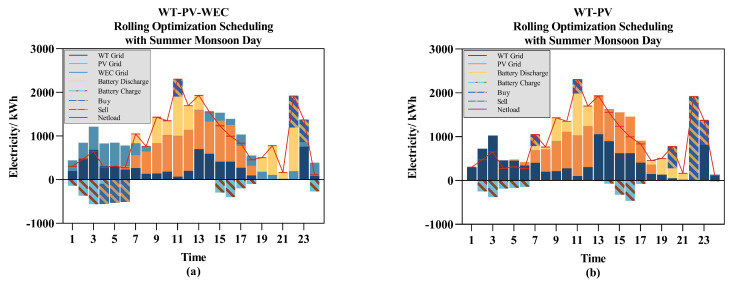
The impact of the WEC microgrid on the scheduling system: (**a**) WT-PV-WEC; (**b**) WT-PV.

Figure 11 presents the scheduling plans in two different scenarios. As observed from the figure, despite the consistency in installed capacity of the power generation system, ESS capacity, etc., across both simulations, the inclusion of WEC resulted in a greater reserve of electricity. In Figure 11a, there is surplus electricity that is sold back to the main grid, further reducing costs. Comparing periods 21:00–23:00 in Figure 11a,b, the system with WEC still has enough energy in the ESS to cover the day’s energy shortfall, whereas the system without WEC has to purchase more expensive electricity from the main grid.

Table 5 compares the operating costs from two simulation experiments, demonstrating that the system incorporating WT, PV, and WEC has an operating cost that is 5% lower than the system solely comprising WT and PV.

Then, we compared the system costs of using only day-ahead optimization with those of employing both day-ahead and intra-day rolling optimization. According to the description in the previous section, the system costs are represented as follows:

(22) $$C_{System}=\left\{\begin{array}{l} \sum_{i}^{N}\sum_{t}^{T}\left(C_{t,i}^{DG}+C_{t}^{R}+C_{t}^{ESS}+C_{t}^{E}+C_{t}^{deficit}+C_{t}^{abandon}),(a\right)\\ \sum_{i}^{N}\sum_{t}^{T}\left(C_{t,i}^{DG}+C_{t}^{R}+C_{t}^{ESS}+C_{t}^{E}+C_{t}^{IN}+C_{t}^{deficit}+C_{t}^{abandon}\right),(b) \end{array}\right.$$

**Figure 12:**
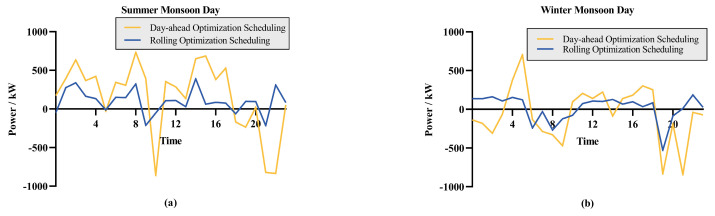
Power deficit and power curtailment scenarios on different typical days: (**a**) the result of summer monsoon days; (**b**) the result of winter monsoon days.

(a) and (b) are Day-ahead optimization and rolling optimization system costs, respectively. Figure 12 illustrates the curtailment and deficit situations within the same typical day for the two optimization strategies, where values greater than 0 represent power curtailment and values less than 0 indicate power deficit. Figure 12a represents the scheduling results for a typical summer monsoon day; Figure 12b represents the scheduling results for a typical winter monsoon day. The system employing the day-ahead and intra-day rolling optimization strategy exhibits significantly lower power curtailment and deficits compared to the system that only uses day-ahead optimization. As a result, the costs associated with $C_{t}^{abandon}$ and $C_{t}^{deficit}$ are significantly higher for the day-ahead optimization alone, leading to increased overall costs. As shown in Table 6, the system costs using rolling optimization scheduling are reduced by 16.1% and 22% on typical winter and summer days, respectively, compared to using day-ahead scheduling.

## 5. Conclusions

This paper proposes a multi-time-scale rolling optimization scheduling model that integrates offshore wind, wave, and photovoltaic energy sources in the Bohai and Yellow Sea regions. This model can enhance grid stability and mitigate the impacts of renewable energy variability on the distribution system by leveraging the complementary characteristics of these energy sources.

Through an in-depth analysis of the collected dataset on renewable energy generation in the test maritime area, we confirmed the complementarity between wind, photovoltaic, and wave energies. Using a CNN-LSTM network, we predicted the power generation for both the day ahead (24 h) and intra-day (1 h), capturing short-term and long-term trends effectively. Subsequently, the DDPG algorithm was employed to explore the predicted state space and identify the optimal scheduling strategy.

Simulation experiments simulated the system’s performance in various monsoonal conditions. The results demonstrate that day-ahead and intra-day rolling optimization can effectively balance power fluctuations through timely intra-day adjustments. The application of energy storage systems (ESSs) also bolstered the system’s capability to cope with renewable energy fluctuations, reducing electricity purchase costs from the grid. Horizontal and vertical analyses showed that rolling optimization scheduling reduces curtailment and power deficits more effectively than traditional day-ahead scheduling, thereby lowering the overall system costs. On typical summer and winter days, the costs of systems using rolling optimization scheduling decreased by 16.1% and 22%, respectively. This study offers valuable insights for the efficient management and optimization scheduling of renewable energy grids in other maritime regions.

## Figures and Tables

**Table 1:** The Kendall coefficient among the three energy sources.

Time	$\tau_{PV-WEC}$	$\tau_{WT-WEC}$	$\tau_{WT-PV}$	$\tau_{(WT+WEC)-PV}$
Annual	−0.5086	0.6114	−0.3649	−0.4106

**Table 2:** Time-of-use (TOU) pricing.

Period	Time Slot	Price (kWh/¥)
Heating period 11.1–3.31 (next year)	Peak Hours 8:00–22:00	0.5769
	Off-Peak Hours 22:00–8:00 (next day)	0.3469
Non-heating period 4.1–10.31	Peak Hours 8:00–20:00	0.5769
	Off-Peak Hours 20:00–8:00 (next day)	0.3769

**Table 3:** The specific values of a, b, and c.

Units	$a_{i}$	$b_{i}$	$c_{i}$
$DG_{i}$	0.0004	0.1	0.15

**Table 4:** The values of parameters.

Parameters	Value	Parameters	Value
$k_{WT}$	0.3	$k_{ESS}$	0.3
$k_{PV}$	0.1	$k_{deficit}$	0.8
$k_{WEC}$	0.2	$k_{abandon}$	0.8

**Table 5:** The costs of WT-PV-WEC and WT-PV.

Scenarios	Operating Cost
WT-PV-WEC	7888.49
WT-PV	8198.15

**Table 6:** The costs of optimization scheduling.

Seasonality	Operating Cost	Penalty Cost	Total Cost
Day-ahead Optimization (Summer)	7888.49	5094.72	12,983.21
Rolling Optimization (Summer)	7253.11	2871.85	10,124.96
Day-ahead Optimization (Winter)	6489.58	5279.33	11,768.91
Rolling Optimization (Winter)	7400.75	2470.31	9871.06

## Data Availability

The data presented in this study are available on request from the corresponding author.
